# Atypical Streptococcal Meningitis with Fatal Cerebrovascular Complications: A Case Report

**DOI:** 10.3390/idr12030018

**Published:** 2020-11-21

**Authors:** Gabriel D. Pinilla-Monsalve, Daniel F. Torres-Cutiva, Juan P. Fernández-Cubillos

**Affiliations:** 1 Facultad de Ciencias de la Salud, Icesi University, Calle 18 No. 122–135, Cali 760032, Colombia; gd.pinilla@gmail.com (G.D.P.-M.); dfelipetorresc@gmail.com (D.F.T.-C.); 2 Department of Neurology, Fundación Valle del Lili, Cra. 98 No. 18–49, Cali 760032, Colombia; 3 Department of Internal Medicine, Fundación Valle del Lili, Cra. 98 No. 18–49, Cali 760032, Colombia

**Keywords:** bacterial meningitis, cranial sinus thrombosis, stroke, *Streptococcus constellatus*, MeSH

## Abstract

Bacterial meningitis is an infectious pathology that remains a public health challenge. The most frequent etiological agent is *Streptococcus pneumoniae*, which is also associated with higher rates of mortality and sequels. However, less is known about the clinical presentation of atypical non-*pneumoniae* streptococcal meningitis. Here, we studied a 23-year-old man with no medical background who presented with projectile vomiting, states of consciousness alteration, unilateral cranial nerve palsy, and meningeal signs. Neuroimaging showed tonsillar herniation, regions of empyema, right transverse and sigmoid sinuses thrombosis, and multiple arterial subcortical infarcts. Cerebrospinal fluid suggested bacterial infection; blood and abscess cultures were positive for *Streptococcus constellatus.* The patient received antibiotics with no clinical improvement. He deteriorated over the following days, the abolishment of brainstem reflexes was observed, and brain death was declared. Streptococcal meningitis produced by atypical species is a potential cause of lethal cerebrovascular complications, even in immunocompetent patients.

## 1. Introduction

Based on the Global Burden of Disease study, cases of meningitis are concentrated in developing countries and represent a threat to public health by producing 280,000 deaths/year even in the era of antibiotics. In Colombia, 20,000 cases are reported yearly, but the real incidence is unclear because of a suspected epidemiological silence. In comparison with global incidence tendencies, which exhibited a marked decrease between 1990 and 2015, the number of new cases per year in this country has remained stable [1]. Most of the Colombian patients with confirmed bacterial meningitis are non-infants, and *S. pneumoniae* is the most common agent. Other microorganisms’ incidence varies according to the patient’s age and vaccination status [2]. In this sense, conjugated vaccines have been widely used with a decrease in the incidence by guaranteeing a sustained immunological response [3]. 

Nonetheless, disability and complications still have a high prevalence, occurring in up to 1 out of 3 patients, and include hydrocephalus, abscesses, cerebritis or cranial nerve involvement, venous thrombosis, arterial infarcts, ventriculitis, and/or extra-axial collections (HACTIVE) [4]. Bacterial meningitis caused by *Streptococcus* species has been correlated with highly disabling sequelae as consequences of subdural empyema, hemorrhages, or ischemia. Indeed, cerebrovascular complications are events of low-to-moderate frequency and mainly affect the cerebral arterial tree [5].

Here, we report a rare and fatal case of venous and arterial thromboses in an immunocompetent patient with meningitis, whose cultures were positive for *S. constellatus*. 

## 2. Case Report

This case report is about a 23-year-old young male, with no relevant background, who attended our Emergency Department by referral from a rural hospital. Eight days before, he complained of right hemifacial pain to his general practitioner who requested basic hematological tests (hemogram and C reactive protein levels) that were apparently normal. He was treated for an upper-airway respiratory viral infection; metamizole and other unspecified anti-inflammatory drugs were prescribed with mild recovery. 

After three days, he presented with hemicranial cephalalgia and subjective fever, accompanied by nausea and several episodes of projectile vomiting which worsened in the following 24 h with an altered state of consciousness. He was taken to the nearest hospital, where he was found with a Glasgow Coma Scale score (GCS) of 3/15 and therefore required orotracheal intubation. Subsequently, he presented with cardiac arrest of an unknown rhythm. Due to his critical state, he was transferred to our Clinic. 

At the Emergency Department, he was febrile (41 °C), tachycardic (102 bpm), and hypotensive (98/48 mm Hg), which was interpreted as a septic state, and aggressive intravenous fluid therapy was initiated. GCS remained impaired and anisocoria was observed, with right mydriasis (5 mm). Emergency physicians requested brain computed tomography which demonstrated diffuse brain edema, bilateral cerebellar tonsil (8 mm), and right uncal herniations. Acute inflammation of the bilateral frontal, left maxillary, and sphenoid sinuses was noted as well. Based on the clinical manifestations, antibiotics were given as per the institutional protocol with a concomitant synthetic steroid (dexamethasone, 8 mg qid).

Due to the suspected intracranial hypertension syndrome, he required hyperventilation and was started on hypertonic saline before performing a lumbar puncture. A small amount of turbid xanthochromic cerebrospinal fluid (CSF) was obtained with pleocytosis (362 white blood cells, 43% neutrophils; 9000 red blood cells) and hyperproteinorrachia (137.4 mg/dL), but with normal glucose levels (76.5 mg/dL). Lactate dehydrogenase concentration (104 U/L) was increased. The patient was transferred to the Intensive Care Unit for neuroprotective measures and the antibiotics scheme was adjusted to ceftriaxone (2 g bid) and vancomycin (1 g bid).

**Figure 1:**
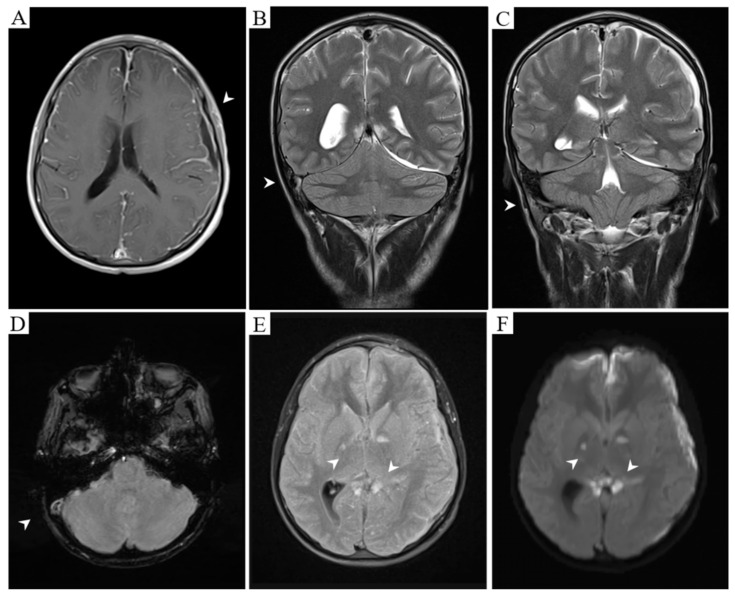
Meningeal enhancement and left fronto-temporo-parietal empyema. Empty delta sign is also observed (**A**, T1 post-gadolinium). Right transverse (**B**, T2) and sigmoid (**C**, T2) sinuses occlusion due to an acute thrombus (**D**, Gradient echo). Established ischemia affecting bilateral globus pallidus and corpus callosum splenium (**E**, T2 FLAIR) with diffusion restriction (**F**, DWI).

On the initial evaluation by Neurology, the patient was under sedative medications, intubated, with right oculomotor palsy (mydriasis and eye abduction in the primary position of gaze) and neck stiffness. A magnetic resonance imaging (MRI) was required and showed the bilateral globus pallidus, internal capsule, corpus callosum, and left uncal ischemic regions. There was empyema in the left fronto-temporo-parietal and right frontal regions with lepto- and pachymeningeal enhancement. Furthermore, angio-MRI was highly suggestive of right transverse and sigmoid venous sinuses thrombosis (Figure 1).

He underwent neurosurgical drainage of 100 milliliters of purulent material; adjuvant antibiotic therapy was initiated with metronidazole (500 mg tid). Subdural empyema was positive for *Streptococcus constellatus,* and the same germ was obtained from repeated blood cultures. Hence, antibiotic regimens were discontinued, except for ceftriaxone, according to the antimicrobial susceptibility profile. Anticoagulant therapy was not defined, considering the major surgical procedure that was performed. 

Unfortunately, his neurological state did not improve, and he developed episodes of severe bradycardia in the postoperative period. Vasopressors were initiated for refractory hypotension. On the seventh day, there was no respiratory effort and decerebrate posturing with brainstem reflexes’ abolishment was recognized. An apnea test was ordered on the next day; results were compatible with encephalic death. 

## 3. Discussion

Acute bacterial meningitis is an inflammatory process of the meningeal layers and subarachnoid space with or without the involvement of the brain or spinal cord parenchyma due to physical proximity. Regardless of the age group, central nervous system bacterial infections of late treatment produce high lethality, specifically whenever streptococcus is isolated (29.77% of deaths are attributable to this germ) [6]. 

For the development of meningitis, *Streptococcus* prototypically colonizes the nasopharyngeal mucosa through adhesion to the epithelium, a process that depends on the interaction of surface polypeptides with laminin, platelet-activating factors, and human polymeric immunoglobulin receptors. Afterward, cocci invade the bloodstream and express lipoproteins, which interferes with the complement system. Neuraminidase and pilus-related adhesine facilitate blood-brain barrier translocation, while the production of reactive oxygen species leads to irreversible endothelial cell lesion [7]. Nonetheless, it is known that meningitis can occur in the absence of bacteremia when para-meningeal foci infection is observed [4]. 

Once bacteria enter the subarachnoid space, its exponential growth induces an immunological response orchestrated by microglia, increasing TNF-α, interleukin-1, and 6 concentrations that ends up favoring blood-brain barrier permeability. In this scenario, neutrophils diapedesis is followed by the production of extracellular matrix metalloproteinases, enhancing inflammatory damage [7]. Secondary to meningitis, patients may develop vasospasm with small-vessels vasculitis, producing arterioles occlusion or venous thrombosis [5], with cranial nerve compromise due to mechanical stress derived from brain tissue and pia mater edema [4]. Diffuse cerebral intravascular coagulation significantly contributes to the pathophysiology of infarctions as well [8]. 

Considering the pathophysiology, bacterial meningitis is related to complications, as stated above, including intracranial hemorrhages, arterial stroke, and venous thrombosis. Other systemic processes, such as acute respiratory distress syndrome, disseminated intravascular coagulation, and septic shock might be present [9]. Van de Beek’s group has suggested that the activation of pro-thrombotic factors and inhibition of fibrinolytic substances is the key mechanism behind stroke following streptococcal meningitis, with no findings compatible with inflammation of large-vessels walls. Curiously, venous thrombosis was identified mainly in patients with concomitant arterial occlusion [8]. On the other hand, delayed cerebrovascular injury in the setting of meningitis has been associated with streptococci, apparently with an augmented risk when patients are prescribed steroids [10]. According to Gallegos, this rare complication (around 1% of cases) is depicted in patients with an apparent good resolution of symptoms, who seven days later develop seizures or altered state of consciousness; activation of the complement system, particularly, higher concentrations of C5a and sC5b-9 fractions seem to be involved. For instance, one of the patients reported by the aforementioned author presented with frontal cortical vein thrombosis 12 days after the initial admission for meningeal signs [11].

Kastenbauer et al. studied a series of German patients with pneumococcal meningitis from 1984 to 2002; there were 19 cases of arterial occlusion and 8 venous thromboses, one of which had consequent intracerebral hemorrhage [12]. These authors discussed that a relatively low leucocyte count is associated with intracranial complications, such as stroke, which seems coherent with CSF cytochemical results of this case that also point to a para-meningeal process. After cortical veins [5], cavernous sinuses are the most commonly affected, according to a paper by Bodur and colleagues in 2002 [13]. Furthermore, Tibussek collected over five years the clinical charts of 14 children with late-onset group B streptococcal meningitis who presented cerebrovascular complications, including two with simultaneous ischemic arterial stroke and cerebral venous thrombosis. In most patients with thrombosis and infection by serotype III streptococci, molecular tests for surface-anchored adhesine (hvgA) were positive [14]. 

Complementarily, *S. constellatus* belongs to the *milleri* subgroup and is a variety of *Streptococcus viridans.* It is a commensal microorganism from the oral cavity of relevance in immunocompromised patients with pulmonary, gastrointestinal, and genitourinary purulent infections [15]. It can be isolated from abscesses because acid media favors its proliferation [16]. Some studies have proposed that, as sulfur-reducing bacteria, these microbes produce hydrogen sulfide (H_2_S) from l-cysteine through the action of cystathionine β-synthase to avoid phagocytosis, therefore facilitating abscess formation. This gas is diffused to adjacent tissues, inducing mitochondrial dysfunction on neurons by the reversible inhibition of cytochrome C oxidase and ATP depletion [17]. Moreover, some species from the *viridans* group express streptolysin S, another virulence factor that explains systemic cytotoxicity [18]. For this patient, thrombosis, empyema, and cytotoxicity might have contributed to brain edema and intracranial hypertension, leading to hemodynamic disturbances, which were worsened by septic shock. 

In the case series reviewed by the Danish team of Møller et al., 26 patients were admitted between 1978 and 1998 suffering from acute streptococcal meningitis caused by non-*pneumoniae* species, representing 1.9% of all bacterial etiologies [19]. In most cases, there were predisposing factors for the infection (73.03%). The percentage of patients with brain empyema was 19.23%, but only in two cases (7.69%) were isolated *S. constellatus*. They reported a case with cerebral thrombosis, but did not specify the affected vessel nor the germ [19]. Until 2006, only two immunocompetent patients with meningitis caused by this germ had been reported, as discussed by Bringas-Bollada [20].

A search on PubMed regarding sinuses thrombosis and *S. milleri* species found only 27 cases. Including this report, 57.14% were male patients with a median age of 42 years (IQR 22–54). The most frequent species were *S. constellatus* (42.85%), followed by *S. milleri* (unspecified) (21.42%). Eight cases included information about CSF, which was characterized in six by neutrophilic (77.5%, IQR 51.3–79.8) pleocytosis (289 cells/μL, IQR 106.5–1042.3), normoglycorrachia (43.2 mg/dL, IQR 23.3–61.3), and hyperproteinorrachia (179.7 mg/dL, IQR 72.6–238.7). Of the patients, 57.14% showed abscess or empyema, and the most frequent thromboses were found on the cavernous (71.42%) and transverse (25.00%) sinuses. Among the reports, ceftriaxone was administered in 11 cases, and metronidazole in 10. Anticoagulation was not started in 42.85%, and death was reported only in this case, with another presumably as the result of non-surgically treated brain herniation (Table 1).

Treatment for these infections depends on the focus; chiefly, ceftriaxone for at least two weeks is the antibiotic of excellence, thanks to its distribution in the neural tissue. Intravenous metronidazole and clindamycin can be simultaneously prescribed for up to six weeks when anaerobic bacteria is found in polymicrobial abscesses. Vancomycin is the option when resistance or allergies are evidenced [21]. In the presence of venous thrombosis, anticoagulation has been debatable (the risk might higher than the benefit), but heparins [22] with a possible transition to coumarin derivatives could be useful, as shown in different case reports. 

This case is infrequent in terms of non-immunosuppression history, multiple cerebrovascular complications (both venous and arterial), and the isolated microorganism from blood cultures and empyema (*Streptococcus constellatus*) with a fatal outcome. It is uncertain if this could be a rare case of a delayed injury attributable to the initial anti-inflammatory treatment; nevertheless, the common biphasic course of this entity was not clearly observed, and metamizole was prescribed instead of glucocorticoids. One of the evident conditions that limited our case approach was the advanced state when the patient was transferred to our clinic; in Colombia, several barriers to health services and scarce resources for offering laboratory tests and diagnostic imaging in primary health centers made patients’ access to appropriate diagnoses difficult. Finally, cerebrovascular complications of this case worsened the prognosis and affected brain autoregulation for optimal recovery; therefore, further research in terms of the specific virulence factors associated with septic thrombosis might help with the recognition of high-risk patients. Studies identifying epidemiological and clinical differences between meningitis by *S. pneumoniae* and *S. viridans* group species should also be carried out.

## 4. Conclusions

*S. constellatus* is a saprophyte bacterium from the oral cavity that might produce meningitis in non-immunocompromised patients with para-meningeal foci. It should also be suspected as an etiological agent for brain abscesses and other rare complications, such as cerebrovascular injuries that worsen the functional prognosis and could even lead to death.

## Figures and Tables

**Table 1:** Characterization of cases reported with meningitis and venous sinus thrombosis due to infection by *S. milleri* species. Age (years). Thrombosed sinuses/vein: CS (cavernous), TS (transverse), SGS (sigmoid), SSS (superior sagittal), and/or IJV (internal jugular). Anticoagulant: UFH (unfractionated heparin), LMWH (low-molecular-weight heparin), WARF (warfarin). Full references are provided in Appendix A.

Author (Year)	Country	Age, Sex	Predisposing Factor	Agent	Abscess	Thrombosis	Antimicrobial	Anticoagulant	Sequelae
Myint (1986) [I]	UK	13, M	Orbital cellulitis	*S. milleri*	No	CS	Penicillin, chloramphenicol, metronidazole		Unknown
Pearson (1994) [II]	UK	37, M	Otitis media	*S. milleri*	Yes	TS	Ciprofloxacin, cefuroxime		None
	UK	58, M	Scalp abscess	*S. intermedius*	Yes	TS	Erythromycin, cefuroxime, cefotaxime, etc.		Yes
Blaser (2000) [III]	Switzerland	72, F	Mastoiditis, diabetes	*S. milleri*	Yes	SGS	Ceftriaxone		Unknown
Benhayoun (2003) [IV]	France	13, M	Rhinosinusitis	*S. constellatus*	No	IJV	Metronidazole, cefotaxime, netilmicin	NFH	None
Chang (2003) [V]	Taiwan	39, M	Sinusitis, alcoholism	*S. constellatus*	No	CS	Penicillin		None
Watkins (2003) [VI]	United States	17, F	Intraorbital abscess	*S. milleri*	Yes	CS	Vancomycin, ceftriaxone, metronidazole	WARF	Yes
Ching (2006) [VII]	UK	14, M	Otitis media	*S. milleri*	Yes	SGS	Ceftriaxone and metronidazole		Yes
Hoshino (2006) [VIII]	Japan	56, F	Orbital cellulitis	*S. constellatus*	Yes	CS	Sulbactam/ampicillin		Yes
Pavlovich (2006) [IX]	Canada	55, M	Orbital cellulitis, periodontitis	*S. anginosus*	No	CS	Vancomycin, ceftriaxone, metronidazole, etc.	LMWH	No
Goawalla (2007) [X]	UK	38, M	Orbital cellulitis	*S. constellatus*	Yes	CS	Cefuroxime, metronidazole, amoxicillin, etc.	Unknown	Yes
Udaondo (2008) [XI]	Spain	51, F	Orbital cellulitis	*S. constellatus*	Yes	CS	Ceftriaxone, vancomycin, metronidazole	NFH/LWMH	Yes
Jones (2009) [XII]	New Zealand	54, F	Dental abscess, RA	*S. constellatus*	Yes	CS	Meropenem, clindamycin	LMWH/WARF	Yes
Gonzalez (2012) [XIII]	United States	26, M	Sinusitis, substance abuse	*S. intermedius*	No	CS	Broad-spectrum antibiotics	LMWH	Unknown
Imholz (2012) [XIV]	Switzerland	45, M	Orbital cellulitis, periodontitis	*S. constellatus*	No	CS	Ceftriaxone, vancomycin	LMWH	None
Noel (2013) [XV]	United States	12, M	Sinusitis	*S. constellatus*	No	CS	Broad-spectrum antibiotics, ceftriaxone		Unknown
Sakaida (2012) [XVI]	Japan	75, F	Sinusitis	*S. constellatus*	No	CS	Unknown	NFH	None
Suzuki (2014) [XVII]	Japan	50, F	Otitis media, gingivitis	*S. intermedius*	Yes	TS	Meropenem, vancomycin	Heparin	Yes
Karssemakers (2015) [XVIII]	Netherlands	54, M	Dental caries, conjunctivitis	*S. anginosus*	Yes	CS	Unknown		None
Selvitop (2015) [XIX]	United States	10, F	Mastoiditis, Crohn’s disease	*S. anginosus*	No	CS/SGS/TS	Ceftriaxone	LMWH	None
Shams (2016) [XX]	United States	73, F	Sinusitis	*S. anginosus*	No	CS/TS	Vancomycin, aztreonam, clindamycin, etc.	LMWH/WARF	Yes
Allegrini (2017) [XXI]	Italy	46, F	Dental caries, orbital cellulitis	*S. constellatus*	Yes	CS	Piperacillin/tazobactam	Heparin	None
López (2016) [XXII]	Spain	24, F	Pregnancy, orbital abscess	*S. milleri*	Yes	CS	Meropenem	LMWH/WARF	Yes
Martel (2018) [XXIII]	France	86, M	Sinusitis	*S. intermedius*	No	CS/IJV	Amoxicillin/clavulanic acid	Unknown	None
Branson (2018) [XXIV]	United States	12, M	Sinusitis, orbital cellulitis	*S. anginosus*	Yes	CS/IJV	Meropenem, vancomycin	NFH/LWMH	None
Deliran (2018) [XXV]	Netherlands	38, F	Periodontitis, dental caries	*S. intermedius*	Yes	CS	Ceftriaxone, metronidazole, penicillin, etc.		Yes
Mo (2018) [XXVI]	China	48, M	Alcoholism	*S. constellatus*	No	SSS/TS	Ceftriaxone		Unknown
Pinilla-Torres (2020) [XXVII]	Colombia	23, M	Sinusitis	*S. constellatus*	Yes	SGS/TS	Ceftriaxone, vancomycin, metronidazole		Death

## Appendix A. Full References of Case Reports Included in Table 1

- Myint S. Cavernous sinus thrombosis due to Streptococcus milleri. J Infect. 1986;13:202–3.
- Pearson CR, Riden DK, Garth RJ, Thomas MR. Two cases of lateral sinus thrombosis presenting with extracranial head and neck abscesses. J Laryngol Otol. 1994;108:779–82.
- Blaser B, Greusing B, Häusler R. Bezold’s abscess with wide extension to the lateral skull base. Schweiz Med Wochenschr. 2000;Suppl 125:23S–26S.
- Benhayoun M, Llor J, Van-Den-Abbeele T, Elmaleh M, Mariani P, Beaufils F, et al. Bilateral jugular thrombosis in Lemierre syndrome. Arch Pediatr. 2003;10:1071–4.
- Chang W-N, Chen S-D, Lui C-C, Huang C-R, Lu C-H. Septic cavernous sinus thrombosis due to Streptococcus constellatus infection. J Formos Med Assoc. 2003;102:733–6.
- Watkins LM, Pasternack MS, Banks M, Kousoubris P, Rubin PAD. Bilateral cavernous sinus thromboses and intraorbital abscesses secondary to Streptococcus milleri. Ophthalmology. 2003;110:569–74.
- Ching H-Y, Ramsden JD, Bottrill I. A unique presentation: Bezold’s abscess and glomerulonephritis. Eur J Pediatr. 2006;165:569–70.
- Hoshino C, Satoh N, Sugawara S, Kuriyama C, Kikuchi A, Ohta M. Septic cavernous sinus thrombosis complicated by narrowing of the internal carotid artery, subarachnoid abscess and multiple pulmonary septic emboli. Intern Med. 2007;46:317–23.
- Pavlovich P, Looi A, Rootman J. Septic thrombosis of the cavernous sinus: two different mechanisms. Orbit. 2006;25:39–43.
- Goawalla A, Mansell N, Pearson A. Septic cavernous sinus thrombosis with bilateral secondary orbital infection. Orbit. 2007;26:113–6.
- Udaondo P, Garcia-Delpech S, Díaz-Llopis M, Salom D, Garcia-Pous M, Strottmann JM. Bilateral intraorbital abscesses and cavernous sinus thromboses secondary to Streptococcus milleri with a favorable outcome. Ophthal Plast Reconstr Surg. 2008;24:408–10.
- Jones RG, Arnold B. Sudden onset proptosis secondary to cavernous sinus thrombosis from underlying mandibular dental infection. BMJ Case Rep. 2009;2009:bcr0320091671.
- Gonzalez L, Holznecth C, Apoorv B. Not a typical sinus infection. In: ACP Clinical Vignette Presentations. Medical College of Wisconsin; 2012. p. 1–22.
- Imholz B, Becker M, Lombardi T, Scolozzi P. Septic thrombosis of the cavernous sinus secondary to a Streptococcus milleri oral infection. Dentomaxillofac Radiol. 2012;41:525–8.
- Noel N, Pandit R, Kale T, Jacobs N. A 13-year-old male with ptosis. Pediatr Ann. 2013;42:61–3.
- Sakaida H, Kobayashi M, Ito A, Takeuchi K. Cavernous sinus thrombosis: linking a swollen red eye and headache. Lancet. 2014;384:928.
- Suzuki Y, Ogawa K, Oishi M, Kamei S. A case of Streptococcus intermedius-induced subdural abscess and left transverse sinus thrombosis occurring subsequent to treatment for gingivitis. Neurology Asia. 2014;19:405–7.
- Karssemakers LHE, Forouzanfar T, Schulten EAJM, Karagozoglu KH. Bilateral conjunctival swelling after dental extraction. Lancet Infect Dis. 2015;15:746.
- Selvitop O, Poretti A, Huisman TA, Wagner MW. Cerebral sinovenous thrombosis in a child with Crohn’s disease, otitis media, and meningitis. Neuroradiol J. 2015;28:274–7.
- Shams PN, Policeni B, Carter KD, Shriver E, Thurtell MJ. Bilateral septic cavernous sinus thrombosis, congestive orbitopathy, and ischemic optic neuropathy. Can J Ophthalmol. 2016;51:e75–7.
- Allegrini D, Reposi S, Nocerino E, Pece A. Odontogenic orbital cellulitis associated with cavernous sinus thrombosis and pulmonary embolism: a case report. J Med Case Rep. 2017;11:164.
- López F, Santamarta E, Martínez P, Sáiz-Ayala A, Llorente JL. Cavernous sinus thrombosis during pregnancy. Auris Nasus Larynx. 2017;44:232–6.
- Martel A. Septic thrombosis of cavernous sinus extended to the ipsilateral internal jugular vein and transversal sinus with favorable outcome: Clinical and radiological features of a Lemierre syndrome. Orbit. 2018;37:94–6.
- Branson SV, McClintic E, Yeatts RP. Septic cavernous sinus thrombosis associated with orbital cellulitis: A report of 6 cases and review of literature. Ophthal Plast Reconstr Surg. 2018.
- Deliran S, Sondag L, Leijten Q, Tuladhar A, Meijer F. Hoofdpijn: denk aan sinus-cavernosus tromboflebitis. Ned Tijdschr Geneeskd. 2018;162:D2907.
- Mo S, Wei L, Chen H, Li R, Li S, Luo G. A Chinese case of Prevotella intermedia and Streptococcus constellatus intracranial mixed infection. Metab Brain Dis. 2018;33:161–6.
